# Depression and Anxiety Symptoms “Among the Waves” of the COVID-19 Pandemic in Obsessive Compulsive Disorder and Adjustment Disorder Patients

**DOI:** 10.3390/healthcare11091261

**Published:** 2023-04-28

**Authors:** Giordano D’Urso, Mattia Vittorio Pomes, Alfonso Magliacano, Carla Iuliano, Hekla Lamberti, Marco Manzo, Teresa Sissy Mariniello, Felice Iasevoli, Andrea de Bartolomeis

**Affiliations:** 1 Section of Psychiatry, Department of Neuroscience, Reproductive and Odontostomatological Sciences, University of Naples Federico II, 80131 Naples, Italy; 2 IRCCS Fondazione Don Carlo Gnocchi ONLUS, 50143 Florence, Italy; 3 School of Cognitive Psychotherapy (SPC), 80100 Naples, Italy

**Keywords:** COVID-19, obsessive compulsive disorder, adjustment disorder, depression, anxiety

## Abstract

The COVID-19 pandemic and the associated restrictions caused great psychological suffering to the general population and psychiatric patients. We aimed to explore the course of depression and anxiety symptoms in obsessive compulsive disorder (OCD) patients, adjustment disorder (AD) patients, and participants without psychiatric disorders (control group, CG) across the different phases of the pandemic: the first lockdown, a temporary interruption of restrictions, and the second lockdown. Out of the 158 patients screened, we enrolled 46 OCD and 19 AD patients as well as 29 CG participants. The Beck Depression Inventory-II and the State-Trait Anxiety Inventory-Y were administered to all participants at each time point. The results showed different symptom severities among the groups throughout the whole study, with OCD patients always scoring higher than AD patients and the CG, and the AD patients always scoring higher than the CG. The symptom course within each group was different. OCD patients’ symptoms sharply worsened during the first lockdown and then remained stable irrespective of the subsequent pandemic phases. In the AD and CG groups, symptoms waxed and waned following the fluctuations of the restriction provisions, with a complete return to the baseline when the restrictions were stopped only in the CG. These findings suggest that the influence of the pandemic and of the associated restrictions on depression and anxiety manifestations may vary depending on the particular pre-existing mental health status.

## 1. Introduction

Coronavirus Disease-19 (COVID-19) is a highly contagious respiratory disease first reported in Wuhan (China) in late 2019. On 11 March 2020, the World Health Organization (WHO) declared a pandemic status [1]. To prevent contagion, WHO has drawn up hygiene-related guidelines including social distancing, mask use, and frequent handwashing, while many governments have instituted lockdowns as preventive measures [2]. Because of the fear of contagion, restrictions, and the quarantine-related psychological distress, the COVID-19 pandemic has had a dramatic impact on the mental health of the general population and of psychiatric patients [3,4,5,6]. Italy was the first European country to experience a severe outbreak of the disease, with the first confirmed case of infection dating to 20 February 2020.

Fear of infection, and frequent handwashing and cleaning are typical symptoms of obsessive compulsive disorder (OCD), a mental disorder characterized by the presence of obsessions and/or compulsions. Obsessions are defined as unwanted thoughts, images, and impulses that occur involuntarily in the mind of an individual and cause them anxiety, while compulsions are defined as repeated rituals in the form of behaviors or mental actions, whose purpose is generally the relief from the anxiety caused by the obsessions. In a great proportion of OCD patients, the obsessions are related to the fear of contamination, and the compulsions consist of repeated washing and cleaning [7].

The COVID-19 pandemic-related psychological distress was strongly associated with the risk of contagion and with the urge to apply strict preventive health rules. For this reason, patients with OCD could have been affected more than the general population and, probably, more than patients with other mental disorders [8].

Adjustment disorder (AD) is defined as having developed emotional or behavioral symptoms out of proportion to the severity or intensity of an identifiable stressor [9]. The symptoms of AD usually consist of anxiety and depression but can also include conduct disturbances.

According to the fluctuations in the infection rates as well as to the economic urges, the health authorities adopted alternating restriction measures, which probably became an additional source of emotional distress both for the general population and psychiatric patients.

The article by Rossi et al. (2020) provided a starting point for reflection on the impact of the pandemic and related restrictive measures on the general population in Italy. The study assessed rates of mental health outcomes in the Italian general population three to four weeks into lockdown measures and explored the impact of COVID-19-related potential risk factors [10]. However, no subsequent reports were published reporting the possible fluctuations of psychiatric symptoms in correspondence to the different degrees of restriction that accompanied the different phases of the pandemic in Italy. Studies conducted in countries with a very different timing of restriction provisions reported that levels of anxiety and depression in the general population increased during the initial phases of lockdown and decreased over the course of weeks [11].

In the present study, we aimed to explore the course of depression and anxiety symptoms in two specifically vulnerable clinical populations, i.e., OCD and AD patients, compared to participants without psychiatric disorders (control group, CG) across different phases of the COVID-19 pandemic. It adds to the existing literature for participants at a higher risk of worsening in consequence of two concurrent peculiar factors. First, the pre-existing psychiatric condition of the patients enrolled (OCD or AD), possibly implying an additional risk due to the specific psychopathological features. Second, the geographical area in which the study took place (the Campania region of Italy), where the restrictions and limitation rules were more precocious in comparison with other countries and more severe and more strictly enforced in comparison with Italian regions other than Campania. This greater strictness was due to the very high population density of the region and to the outstanding consequent risk of infection spread.

Our hypothesis was that the three study groups (OCD, AD, and CG) would exhibit differences in the course of their anxiety and depression symptoms, as measured by specific rating scales. In particular, we hypothesized that at each time point the severity of symptoms would be higher in the OCD group, intermediate in the AD group, and lower in the CG participants. Moreover, we hypothesized that, while symptom severity varied in CG participants in response to changes in pandemic trends (and consequent restrictions), this association was less evident for the AD group and definitely not present in the OCD group.

## 2. Method

### 2.1. Participants

The participants were enrolled and the data were collected for this study at four time points: (1) during the three months before the pandemic outbreak (T0); (2) from 22 April to 18 May 2020, corresponding to the date of the ethical approval of the study by our ethics committee and that of the end of the first mandatory lockdown in Italy, respectively (T1); (3) between June and September 2020, during a temporary elimination of restriction provisions (T2); and (4) in November and December 2020, during the second mandatory lockdown in Italy (T3).

At T0, one hundred and fifty-eight patients were selected from the clinical records of the OCD outpatient clinic and the general psychiatry outpatients clinic of the Psychiatry Unit of the University Hospital “Federico II” in Naples, Italy. Among them, 102 were OCD patients and 56 were AD patients, diagnosed according to DSM-V criteria.

The inclusion criteria were (1) patients who visited during the three months before the pandemic outbreak, and (2) were clinically stable at the time of the last visit (i.e., no change in treatment). The exclusion criterion was the presence of psychiatric comorbidities (i.e., no further psychiatric diagnoses other than OCD and AD, according to DSM-V criteria). Among the screened patients, 46 (45%) OCD and 19 (34%) AD patients agreed to take part in the study, while the remaining refused for personal reasons or were not reachable before the end of the lockdown.

A convenience sample of 29 participants with no AD or OCD (control group, CG) was also enrolled by means of word-of-mouth, according to a snowball sampling method.

### 2.2. Measures

The assessment consisted of administering two rating scales at the four time points. The rating scales used were the Beck Depression Inventory-II (BDI-II) [12] and the State-Trait Anxiety Inventory-Y (STAI-Y) [13]. The BDI-II is a multiple-choice, self-reported inventory consisting of 21 items that assess the affective, cognitive, and physical symptoms of depression. Each item is rated from 0 to 3 (from the least to the most severe). The STAI-Y is a commonly used measure of trait and state anxiety. It includes 20 items for assessing trait anxiety and 20 for state anxiety. For this study, we only used the items assessing state anxiety and not those assessing trait anxiety.

The participants’ demographic characteristics (age, sex, education, illness duration) were also collected.

### 2.3. Procedure

For the OCD and the AD groups, the baseline data were taken from clinical records reporting scores of the above-mentioned scales administered during the most recent (no further than three months earlier) in-person visit, while the follow-up assessments were made during psychiatric visits through video calls.

Only for CG participants, STAI-Y and BDI-II scores at each time point were collected during the video-calls, with the baseline evaluation being retrospective in nature.

### 2.4. Statistical Analyses

After testing for normality (Shapiro–Wilk test) and for homogeneity of variances (Levene’s test), the participants’ characteristics were compared across the groups (OCD, AD, and CG) by means of the Kruskal–Wallis H test (i.e., age), Mann–Whitney U test (i.e., illness duration), Chi-square test (i.e., sex, education), ANOVA (i.e., STAI-Y at T0 and T2), or by means of Welch’s U test (i.e., STAI-Y at T1 and T3, BDI-II at T0, T1, T2, and T3), according to Skovlund and Fenstad [14]. In the case of significant results, pairwise comparisons were performed by means of the Mann–Whitney U test (i.e., age), Chi-square test (i.e., sex, education), *t*-test (i.e., STAI-Y at T0 and T2), or by means of Welch’s U test (i.e., STAI-Y at T1 and T3, BDI-II at T0, T1, T2, and T3).

The scores of the STAI-Y and BDI-II were compared across time points (T0, T1, T2, T3) in each group by means of the Wilcoxon signed-rank test. We performed a post hoc power analysis for Wilcoxon signed-rank tests in order to calculate the achieved power for each study group using G*Power v. 3.1.9.6.

The level of significance was set at 0.05. All analyses were performed using IBM SPSS v. 25 (IBM Corp., Armonk, NY, USA).

### 2.5. Ethics

All procedures were in accordance with the Declaration of Helsinki and its later amendments and were approved by the Ethics Committee for Biomedical Activities of the University of Naples Federico II (approval code 152/20, 22 April 2020). The participants provided their written informed consent by email after a phone interview in which the purposes, procedures, and time points of the longitudinal study were clearly explained.

## 3. Results

We recruited a purposive sample of 94 participants, consisting of 46 patients with OCD, 19 patients with AD, and 29 without OCD or AD (CG; 51 females, median age was 42, IQR = 28 years). All participants were Caucasian.

The three groups showed no significant differences in age, gender, or education level (Table 1).

The patients with OCD (14/46, 30.4%) and AD (15/19, 78.9%) had a higher frequency of medical comorbidities than participants in the control group (5/28, 17.8%; χ^2^ = 19.68; df = 2; *p* < 0.001), with a significant difference also between the OCD and AD groups (χ^2^ = 12.80; df = 1; *p* < 0.001). The percentage of individuals who contracted COVID-19, or worked as health professionals, or were affected economically by the pandemic did not differ significantly across the groups (all *p* > 0.05).

At baseline, the levels of anxiety and of depression differed significantly across the three groups (Table 1). In particular, the OCD group showed significantly higher anxiety and depression than the CG (U = 372.0; *p* = 0.001 and U = 310.0; *p* < 0.001, respectively), but not the AD group (U = 347.5; *p* = 0.196 and U = 395.5; *p* = 0.549, respectively).

The AD group showed significantly higher depression (U = 163.5; *p* = 0.018), but not anxiety (U = 186.5; *p* = 0.060) compared to the CG.

At the following time points, significant differences were found in the levels of anxiety and depression (Table 1).

Specifically, the OCD group showed significantly higher anxiety and depression than the CG (U = 440.5; *p* = 0.014 and U = 277.5; *p* < 0.001, respectively) but not AD group (U = 345.5; *p* = 0.187 and U = 372.5; *p* = 0.352, respectively).

The AD group showed significantly higher depression (U = 162.0; *p* = 0.017) but not anxiety (U = 219.5; *p* = 0.237) compared to the CG.

**Figure 1:**
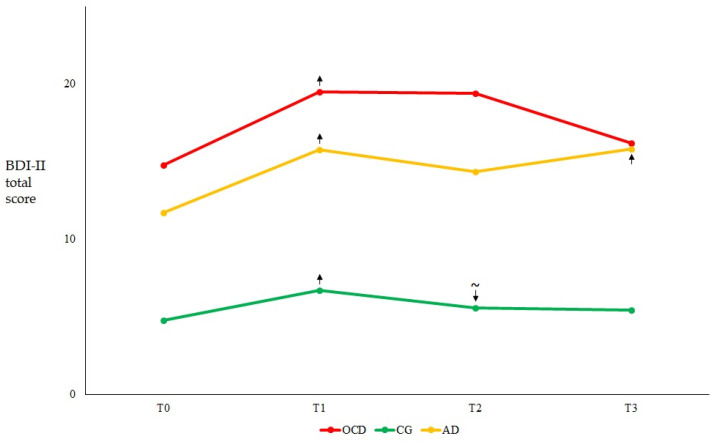
The graph displays the mean BDI-II values for each of the three analyzed groups at the four study time points. Arrows indicate the direction of significant changes with respect to the previous time point. ~indicates a quasi-significant change (*p* = 0.051). Abbreviations: AD = Adjustment Disorder; BDI-II = Beck Depression Inventory-II; CG = Control Group; OCD = Obsessive Compulsive Disorder. T0 = three months before the pandemic outbreak; T1 = during the first mandatory lockdown in Italy; T2 = during a temporary interruption of restriction provisions; T3 = during the second mandatory lockdown in Italy.

**Figure 2:**
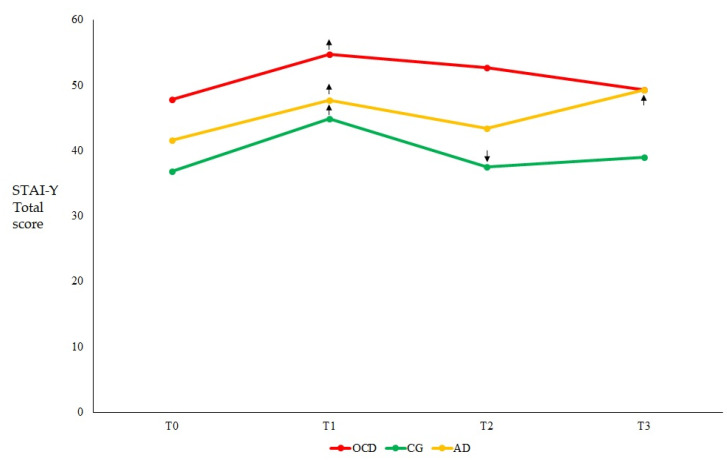
The graph displays the mean STAI-Y values for each of the three groups analyzed at the four study time points. Arrows indicate the direction of significant changes with respect to the previous time point. Abbreviations: AD = Adjustment Disorder; CG = Control Group; OCD = Obsessive Compulsive Disorder; STAI-Y = State-Trait Anxiety Inventory-Y. T0 = three months before the pandemic outbreak; T1 = during the first mandatory lockdown in Italy; T2 = during a temporary interruption of restriction provisions; T3 = during the second mandatory lockdown in Italy.

Overall, depression (Figure 1) and anxiety (Figure 2) were significantly higher at T1 compared to baseline in all groups, with significant increases seen in both measures across all groups and time points (Table 2).

At T2 and T3, significant differences were found across the groups at all time points.

Specifically, anxiety and depression were higher in the OCD and AD groups than in the CG (Table 1).

No significant differences were found between the OCD and AD groups at T2 (all *p* > 0.05; Table 1).

In regard to the symptom course within each group, OCD patients showed stable depression (Figure 1) and anxiety (Figure 2) scores across time after the significant increase of both scores at T1 with respect to T0 (Table 2), although the level of anxiety observed at T3 was significantly lower than that observed at T1 (Z = −2.548; *p* = 0.011). In the AD group, both depression (Figure 1) and anxiety (Figure 2) scores significantly increased at T1 with respect to T0, but remained stable at T2, and then further increased at T3 (compared to both T2 and T0; Table 2). In the CG, both depression and anxiety scores significantly increased at T1 compared to T0; thereafter, anxiety scores significantly decreased to the baseline level at T2 and remained stable at T3 (Figure 2). The same tendency was observed for depression scores (Figure 1), although the decrease between T2 and T1 was approaching statistical significance (Table 2).

## 4. Discussion

The present study aimed to investigate depression and anxiety symptoms in two clinical populations, i.e., OCD and AD patients, compared to participants without OCD or AD across four different phases of the COVID-19 pandemic.

The results point to different symptom severities at all time points, with OCD patients scoring higher than the other two groups and AD patients scoring higher than the CG.

However, the symptom course within each group was different. OCD patients’ depression and anxiety sharply worsened during the first lockdown and then remained stable irrespective of the pandemic phases. In AD patients, depression and anxiety showed a further increase during a second contagion wave. Conversely, in participants without AD or OCD, anxiety and depression increased during the lockdown with respect to the baseline, but waned at the end of the lockdown and then remained stable.

In Italy, two large COVID-19 epidemiological waves occurred in 2020, during the spring and the autumn, accompanied by severe restriction provisions. Between the two waves, i.e., during the summer, the reduction of the infection rate allowed a nearly complete elimination of restrictions, with people being able to resume most of their normal activities. The second wave was less intense than the first and was probably facilitated by the frequent social contacts characterizing the activities of the summer vacation. As we already reported, anxiety and depression symptoms increased during the first lockdown regardless of the pre-existing psychiatric disorder, and this increase was even greater in participants without OCD or AD than in the patients [15]. The present study reports data from the continuation phase of that previous study and focused on the symptom course during the following phases of the COVID-19 pandemic.

In the OCD group, increased anxiety and depression persisted across subsequent time points, suggesting that OCD-specific mechanisms might be involved in maintaining these symptoms beyond the distressing circumstances. Considering the contamination-related symptoms that characterize OCD, the patients with this disorder can be considered an at-risk population during the pandemic. For these reasons, a network of OCD experts published specific guidance to help mental health professionals adjust the treatment of patients with OCD during the pandemic [16]. However, the previous studies reported controversial findings on the topic. On one hand, a recent large-scale online survey of OCD patients reported a worsening of OCD symptoms in 76% of participants during the pandemic [17], while another study showed that patients with OCD either started to display new types of obsessions and compulsions or showed past symptoms that were not present before the COVID-19 outbreak [18]. An increased incidence of symptoms related to contamination was also documented, among which the most often reported were the avoidance behaviors towards possible sources of contagion. Moreover, the remission status and having contamination symptoms before the quarantine were associated with greater symptom worsening [19]. On the other hand, a study on a large cohort of OCD patients found no difference in OCD symptoms prior to vs. during the pandemic [20], and another study found no change in OCD symptoms among children and adolescents [21]. Nevertheless, even if the COVID-19 literature reports controversial findings about OCD-specific symptoms, our study suggests that anxiety and depression of OCD patients did worsen during the pandemic and that this effect was greater than in both AD patients and in persons without psychiatric disorders, probably due to a higher non-specific psychopathological vulnerability.

Although the occurrence of AD could imply a greater vulnerability to any kind of stressors, surprisingly no study specifically investigated the reaction of patients with this disorder to the pandemic-related emotional distress.

In the AD group, the symptoms worsened during the first lockdown (T1), persisted during the temporary interruption of restrictions (T2), and further worsened during the second pandemic wave and consequent lockdown (T3). Apparently, the emotional distress associated with the lockdown triggered a persistent state of alarm, which in turn predisposed to further symptom worsening at the time of the second pandemic wave, even though the severity of the corresponding restrictions was lower than during the first wave. A possible explanation for this is the phenomenon of kindling, consisting in a progressive increase in the severity and frequency of symptoms following repeated exposure to stressors, leading to a state of sensitization and decreased threshold for subsequent stressors. This phenomenon has been observed in various psychiatric conditions, including anxiety and mood disorders, and may be related to neurobiological changes such as alterations in the HPA axis and neurotransmitter systems [22,23]. A more simplistic explanation is that a very strong disappointment occurred at the time of the second wave because the summer “green light” had given everyone the feeling that the alarming situation had passed. Moreover, a previous study suggested that frequent exposure to information regarding the COVID-19 outbreak, fear of contagion, and hypochondriasis symptoms would, in turn, increase individuals’ susceptibility to maladjustment to stressful situations [24]. It should be noted that several profiles of responses to the pandemic have been found in AD patients [25], and thus different explanations might apply to different AD sub-groups. Unfortunately, we could not fully address this possibility here because of the small sample size.

In the CG group, both depression and anxiety scores increased during the first lockdown, although to a lesser extent and starting from lower baseline levels compared to the other two groups. However, unlike both the other groups, symptom scores returned to the baseline during the summer and remained so even during the second pandemic wave. A possible explanation for this different course is that participants without AD and OCD might show greater psychological resources or resilience [26,27], thus having a higher likelihood of exhibiting efficacious coping mechanisms [28]. However, we did not collect measures of resilience in our samples; therefore, this hypothesis should be taken with caution and be confirmed in further studies.

## 5. Limitations

This study has several methodological limitations. Firstly, the small sample size may have hindered our ability to detect significant differences during follow-up, including the progression of OCD symptoms. This was due to the brief time frame between the approval of the ethics committee and the conclusion of the mandatory lockdown in Italy, which resulted in a decrease in the size of the sample. During this period and in the unprecedented circumstances of the lockdown, a considerable number of patients chosen from their medical records were either unreachable or unwilling to participate due to personal or health reasons, leading to a low recruitment rate for the study (45% for the OCD group, 34% for the AD group). Furthermore, the decision not to include patients who had not been visited within three months before the onset of the pandemic also restricted the sample size. This decision was made to eliminate the potential confounding influence of significant life events that may have occurred between a more remote time point and the lockdown. Notwithstanding the small sample size, the results of the post hoc power analyses revealed that our findings are solid, especially in the OCD and CG groups.

Secondly, baseline and follow-up data of the CG were collected within a single video-call during the lockdown, probably affecting baseline data reliability due to physiological oblivion, state-dependent memory recall, and selective attention biases of respondents. Thirdly, we adopted different procedures (i.e., clinical records vs. video-calls) for the collection of baseline data in the OCD, AD, and CG groups. This difference might have influenced the comparison of baseline findings in the three groups.

Moreover, we took into account anxiety and depression, which are non-specific psychiatric symptoms and did not consider disorder-specific symptomatology. This did not allow us to reach a comprehensive conclusion about the impact of pandemic restrictions on these psychiatric disorders. Finally, our results can only be generalized to Caucasian ethnicities, since no other ethnic group was enrolled.

## 6. Implications and Future Directions

Overall, these findings suggest that the COVID-19 lockdown was universally distressing, since an increase in anxiety and depression during the lockdown was observed regardless of mental health status. As a matter of fact, new methods for detecting demographic and clinical indicators of vulnerability to the onset and/or worsening of such widespread symptoms in the case of severe restrictions could help prevent much of the suffering related to such dramatic contingencies [29].

Subsequently, the influence of the pandemic and the associated restrictions on depression and anxiety manifestations varied depending on the particular pre-existing mental health status. Algorithms including risk factors for the development of psychiatric symptoms in emergency conditions should be regularly used in clinical practice to prevent and/or promptly treat such symptoms [2].

Moreover, further research is warranted to understand the psychological and neurobiological mechanisms underlying the vulnerability to psychopathology in highly distressing situations such as the pandemic and the associated restriction measures.

## 7. Conclusions

Our study adds to the growing body of literature suggesting that the COVID-19 pandemic has had a significant impact on vulnerability to anxiety and depression onset and persistence in individuals with pre-existing psychiatric disorders, particularly those with OCD and AD, as well as on that of the general population. According to our findings, OCD patients were at a higher risk of sharp and persisting symptom worsening during the pandemic, compared to the other study groups. However, AD patients showed a more gradual but more progressive symptom worsening, suggesting the presence of sensitization to stressors resulting from repeated exposure. Mental health professionals should be aware of these different responses of the clinical populations in order to implement prevention and treatment strategies accordingly.

## Figures and Tables

**Table 1:** Descriptive statistics of the participants as a function of the study group.

	OCD (*n* = 46)	AD (*n* = 19)	CG (*n* = 29)	Statistics	*p*
Sex (M/F)	24/22	5/14	14/15	χ^2^ = 3.731	0.155
Age (years)	39.6 ± 14.8	49.4 ± 16.5	41.0 ± 15.1	H = 5.266	0.072
Education (primary/middle/high/ bachelor/master)	1/10/21/14	1/8/8/2	0/8/14/7	χ^2^ = 5.788	0.447
Illness duration (months)	200.5 ± 163.8	151.8 ± 100.6	/	U = 391.5	0.511
STAI-Y					
T0	47.9 ± 14.3 ^a^	41.6 ± 13.2	36.9 ± 11.2 ^a^	F = 6.336	**0.003**
T1	54.8 ± 16.3 ^a^	47.7 ± 16.8	44.9 ± 10.8 ^a^	W = 4.988	**0.011**
T2	52.7 ± 15.0 ^a,b^	43.4 ± 14.2 ^b^	37.5 ± 11.0 ^a^	F = 11.418	**<0.001**
T3	49.3 ± 14.8 ^a^	49.3 ± 9.9 ^c^	39.0 ± 11.5 ^a,c^	W = 7.471	**0.001**
BDI-II					
T0	14.8 ± 13.4 ^a^	11.7 ± 9.9 ^c^	4.8 ± 4.9 ^a,c^	W = 12.681	**<0.001**
T1	19.5 ± 14.9 ^a^	15.8.3 ± 12.4 ^c^	6.7 ± 5.5 ^a,c^	W = 16.271	**<0.001**
T2	19.4 ± 15.2 ^a^	14.4 ± 11.2 ^c^	5.6 ± 5.0 ^a,c^	W = 19.060	**<0.001**
T3	16.2 ± 14.9 ^a^	15.8 ± 11.8 ^c^	5.4 ± 5.3 ^a,c^	W = 14.325	**<0.001**

Descriptiv Data are reported as mean ± standard deviation for continuous variables and as counts for categorical variables. Univariate statistics are based upon the Kruskal–Wallis H test, Mann–Whitney U test, Chi-square test, ANOVA, or by means of Welch’s U test, as appropriate. Significant differences across the three groups are reported in bold. ^a,b^ and ^c^ indicate significant differences between two groups. Abbreviations: AD = Adjustment Disorder; BDI-II = Beck Depression Inventory-II; F = Female; CG = Control Group; M = Male; OCD = Obsessive Compulsive Disorder; STAI-Y = State-Trait Anxiety Inventory-Y.

**Table 2:** Statistics of the comparisons across time points for each study group.

	T1-T0	T2-T1	T3-T2
OCD			
STAI-Y	Z = 3.232 (***p* = 0.001**; 86%)	Z = −1.944 (*p* = 0.052; 44%)	Z = −0.884 (*p* = 0.377; 13%)
BDI-II	Z = −2.968 (***p* = 0.003**; 79%)	Z = −0.555 (*p* = 0.579; 8%)	Z = −1.526 (*p* = 0.127; 29%)
AD			
STAI-Y	Z = −2.840 (***p* = 0.005**; 74%)	Z = −1.855 (*p* = 0.064; 39%)	Z = −2.306 (***p* = 0.021**; 56%)
BDI-II	Z = −3.189 (***p* = 0.001**; 83%)	Z = −0.870 (*p* = 0.384; 12%)	Z = −2.076 (***p* = 0.038**; 47%)
CG			
STAI-Y	Z = −3.201 (***p* = 0.001**; 84%)	Z = −3.618 (***p* < 0.001**; 92%)	Z = −1.329 (*p* = 0.184; 24%)
BDI-II	Z = −2.890 (***p* = 0.004**; 78%)	Z = −1.953 (*p* = 0.051; 44%)	Z = −0.312 (*p* = 0.755; 6%)

Statistics are based upon the Wilcoxon signed-rank test. Significant differences across time points are reported in bold. Percentages indicate the achieved statistical power. Abbreviations: AD = Adjustment Disorder; BDI-II = Beck Depression Inventory-II; CG = Control Group; OCD = Obsessive Compulsive Disorder; STAI-Y = State-Trait Anxiety Inventory-Y; T0 = three months before the pandemic outbreak; T1 = during the first mandatory lockdown in Italy; T2 = during a temporary interruption of restriction provisions; T3 = during the second mandatory lockdown in Italy.

## Data Availability

The data presented in this study are available on request from the corresponding author.
